# Validation of Noninvasive Cutaneous Carotenoid Measurements as Nutritional Biomarker Across Body Composition Groups

**DOI:** 10.1002/fsn3.71960

**Published:** 2026-05-29

**Authors:** Franziska Kiem, Sonja Lackner, Nathalie Meier‐Allard, Christa Schimpel, Sabrina Mörkl, Tina Unterweger, Jan Gruber, Herbert Strobl, Sandra Holasek

**Affiliations:** ^1^ Division of Immunology, Otto Loewi Research Center Medical University of Graz Graz Austria; ^2^ Division of Medical Psychology, Psychosomatics and Psychotherapeutic Medicine, Department of Psychiatry, Psychosomatics and Psychotherapeutic Medicine Medical University of Graz Graz Austria

**Keywords:** antioxidants, biomarker, body composition, carotenoids, HPLC, reflection spectroscopy

## Abstract

Carotenoids are plant‐derived bioactive compounds with antioxidant properties and well‐established roles in human health. Circulating carotenoid concentrations are widely used as an objective biomarker of fruit and vegetable intake; however, their assessment by high‐performance liquid chromatography (HPLC) requires invasive sampling and laboratory infrastructure. Reflection spectroscopy–based devices, such as the Veggie Meter and Biozoom, offer rapid, noninvasive alternatives for assessing cutaneous carotenoids (CC), but their validity across different body composition profiles remains insufficiently characterized. In this study, plasma carotenoid concentrations quantified by HPLC were compared with CC measurements obtained using the Veggie Meter and Biozoom in 54 healthy women across three study visits (*n* = 151 observations). Associations between plasma and cutaneous carotenoids were evaluated using Spearman and partial correlation analyses with adjustment for body fat percentage and were further examined using linear mixed‐effects models (LMMs) accounting for repeated measurements including body fat percentage and smoking status as covariates. Total plasma carotenoid concentrations were strongly correlated with CC values measured by Biozoom (*r_s_* (151) = 0.697, *p* < 0.001) and Veggie Meter (*r_s_* (151) = 0.650, *p* < 0.001). Provitamin A carotenoids exhibited the strongest associations, whereas lutein and lycopene showed weaker correlations. All associations remained statistically significant after adjusting for body fat percentage and smoking status. Both plasma and cutaneous carotenoid levels were inversely associated with BMI and obesity‐related measures (all *p* < 0.0033, after Bonferroni correction for multiple comparisons). These findings support the use of reflection spectroscopy–based cutaneous carotenoid measurements as valid, noninvasive nutritional biomarkers of fruit and vegetable–derived carotenoid exposure. Such tools may facilitate dietary assessment and monitoring in nutrition research, clinical practice, and public health settings, provided that body composition is considered when interpreting results.

## Graphical abstract

Cutaneous carotenoid measurements obtained non‐invasively by reflection spectroscopy (Veggie Meter and Biozoom) showed strong correlations with plasma carotenoid concentrations measured by HPLC in women across different BMI categories (Veggie Meter *r*_*s*_(151) = 0.697 and Biozoom *r*_*s*_(151) = 0.650, both *p* < 0.001). These findings support the use of skin carotenoid assessment as a practical, noninvasive biomarker of fruit and vegetable–derived carotenoid status for nutrition research, clinical practice, and public health applications.

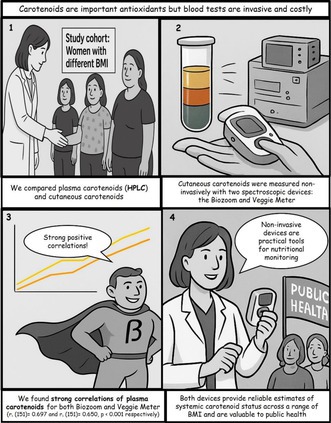

**Abbreviations**
BHT: butylated hydroxytoluene
BIA: bioelectrical impedance analysis
BMI: body mass index
CC: cutaneous carotenoids
EDTA: ethylenediaminetetraacetic acid
ESAN III: Energy Sensing Study III
FFM: fat‐free mass
HPLC: high‐performance liquid chromatography
IQR: interquartile range
LMM: linear mixed‐effects model
NIST: National Institute of Standards and Technology
SD: standard deviation
SPSS: statistical package for the social sciences
SRM: standard reference material
TBF: total body fat
UV–Vis: ultraviolet–visible

## 1 Introduction

Carotenoids are a group of fat‐soluble micronutrients predominantly found in fruit and vegetables. As natural pigments, they are responsible for the vibrant colors of many plants, such as carrots, tomatoes, citrus fruits, and leafy vegetables (Martini et al. 2022). The antioxidant properties of carotenoids are well established, and numerous health benefits have been associated with a diet rich in carotenoids (Bufka et al. 2024; Maiani et al. 2009; Terao 2023). They not only contribute to immune function and visual health but are also inversely associated with chronic conditions such as cardiovascular disease, obesity, and metabolic syndrome (Eggersdorfer and Wyss 2018; Obana 2025). In humans, carotenoids must be obtained from diet; thus, carotenoid levels are considered a reliable biomarker of fruit and vegetable intake and overall nutritional habits (Matsumoto et al. 2020; Mayne et al. 2010; Rush et al. 2020).

From a nutrition science perspective, objective assessment of fruit and vegetable intake remains a major methodological challenge, particularly in intervention studies and population‐based research. Traditional dietary assessment tools, such as food frequency questionnaires, dietary recalls, or food diaries, are prone to reporting and recall bias, misestimation of portion sizes, and underreporting of intake. These limitations highlight the need for valid objective biomarkers of fruit and vegetable intake that can be applied in large‐scale and field‐based settings. Systemic carotenoid levels can be quantified in human plasma via high‐performance liquid chromatography (HPLC) (May et al. 2020). However, HPLC requires invasive blood draws, laboratory infrastructure, and high costs, which limits its feasibility for population‐based or large‐scale studies. In recent years, optical devices based on reflection spectroscopy have emerged as promising alternatives for assessing cutaneous carotenoid (CC) levels. Instruments such as the Veggie Meter and Biozoom measure carotenoid accumulation in the outermost layer of the skin (stratum corneum), offering a rapid, noninvasive, and convenient measurement method (Darvin et al. 2016; Di Noia and Gellermann 2021). These devices were initially validated against skin biopsies and resonance Raman spectroscopy, a highly specific but less accessible reference technique, and have since been applied in nutritional and epidemiological studies (Ermakov et al. 2018; Hwang et al. 2023).

Several studies have demonstrated promising correlations between CC scores and plasma carotenoid levels, supporting the validity of both the Veggie Meter and the Biozoom (Ermakov et al. 2018; Jilcott Pitts et al. 2018; Wu et al. 2025). However, the reliability of these noninvasive tools across different body compositions, particularly in individuals with obesity, remains insufficiently characterized (Hamulka et al. 2023; Meinke et al. 2011). Adipose tissue not only serves as a storage depot for carotenoids but may also sequester them from circulation, complicating their interpretation as biomarkers (Bonet et al. 2016; Hamulka et al. 2023). Higher total body fat (TBF) has been repeatedly associated with lower circulating carotenoid levels, potentially due to increased turnover because of greater inflammation and oxidative stress (Harari et al. 2020). Experimental studies further suggest that oxidative stress accelerates carotenoid depletion, whereas chronic low‐grade inflammation may alter carotenoid metabolism and tissue distribution (Kaulmann and Bohn 2014).

Given these gaps, the present study aimed to evaluate the reliability of spectroscopic CC measurements by comparing them to HPLC‐derived plasma levels in a cohort of healthy women with a wide range of body mass indices (BMIs) and body fat percentages. Additionally, we investigated the relationships between carotenoid concentrations (systemic and cutaneous) and measures of adiposity, including BMI and total body fat (TBF). By analyzing pooled data from a multi‐visit pilot intervention study, independent of treatment effects, we provide robust evidence on whether noninvasive screening of carotenoid status could be reliably applied in clinical nutrition and public health contexts.

## 2 Materials and Methods

### 2.1 Study Population and Design

The present study is part of a larger pilot intervention project on energy sensing (ESAN III) conducted at the Medical University of Graz, Austria. The study was approved by the local ethics committee (No. 36‐273 ex 23/24), registered at ClinicalTrials.gov (NCT06985381) and was performed in accordance with the Declaration of Helsinki. Among the 62 adult women screened, 54 met the eligibility criteria (Table 1). Participants were required to be healthy and free of conditions that could affect carotenoid metabolism or lipid metabolism. Therefore, individuals with lipid metabolism disorders (e.g., hypercholesterolemia or hypertriglyceridemia), diabetes mellitus, gastrointestinal disease, endocrine disorders, immunological disease, or psychiatric disorders were excluded. Additional exclusion criteria included pregnancy or breastfeeding, bariatric surgery, fructose intolerance, acute illness, and alcohol or drug abuse. Participants were grouped based on their BMI, into either normal weight if BMI = 18.5–24.99 kg/m^2^ (*n* = 43) or obese if BMI > 30.0 kg/m^2^ (*n* = 11), according to the WHO definition (Branca et al. 2007). The normal weight group was randomly assigned to either a *verum* or *placebo* arm via the official randomization tool of the Medical University of Graz (www.randomizer.at). The *verum* group was asked to consume 100 mL of a carotenoid‐rich juice twice a day during an intervention period of 6 weeks, whereas the placebo group received water. In the obese group, all participants received juice, and no placebo group was formed because of the low number of participants. The total study period was 12 weeks, consisting of a 6‐week intervention phase followed by a 6‐week washout phase.

**TABLE 1:** Eligibility criteria for the ESAN III study.

Inclusion criteria	Description/value
Sex	Female
Age range [years]	18–65
Health status	Healthy, no known acute or chronic disease
BMI range [kg/m^2^]	18.5–24.9 or > 30.0
Voluntary participation and signed informed consent	True
*Exclusion criteria*	*Examples*
Lipid metabolism disorders	Hypercholesterolemia, hypertriglyceridemia
Metabolic disease	Diabetes mellitus
Bariatric surgery	Previous bariatric procedures
Pregnancy or breastfeeding	Current pregnancy or lactation
Alcohol or drug abuse	Current substance abuse
Acute disease	Fever, cancer, cardiovascular, pulmonological or endocrine disorders
Gastrointestinal disease	Chronic gastrointestinal disorders
Blood disease	Hematological disorders
Immunological disease	Autoimmune or immunological disorders
Psychiatric disorder	Schizophrenia, dementia, bipolar or affective disorders
Endocrine disorder	Hyper‐ or hypothyroidism, hypercortisolism
Fructose intolerance	Diagnosed fructose intolerance

### 2.2 Body Measurements

#### 2.2.1 Anthropometry

At each of the three visits, anthropometric parameters, including height, weight, and waist circumference, were assessed. Height and weight were determined via a digital stadiometer and scale (SECA; Hamburg, Germany), with participants standing upright with their head positioned in the Frankfurt plane. Waist circumference was measured while the subject was standing in a relaxed breathing position at the midpoint between the lowest rib and the top of the iliac crest using a measuring tape.

#### 2.2.2 Bioelectrical Impedance Measurements

Body composition was assessed via bioelectrical impedance (BIA) with the BIA 101 system (Akern Bioresearch; Florence, Italy). The participants were instructed to abstain from intense physical activity and alcohol consumption the evening prior and to arrive in a fasted state with an empty bladder. For the measurement, participants were placed in a lying position, ensuring separate placement of arms and legs. After the skin, adhesive electrodes (BIA Classic Tabs; Medi‐Cal Healthcare; Karlsruhe, Germany) were placed on the dorsal surface of the right hand and foot. Whole‐body impedance was measured at 50 kHz. TBF and fat‐free mass (FFM) were calculated using Bodycomposition V 9.0 software (Medi‐Cal Healthcare) based on the equation of Sun et al. (2003).

### 2.3 Plasma Carotenoids

#### 2.3.1 Sample Collection and Extraction

To obtain plasma, blood was drawn in the morning after a 12‐h overnight fast using EDTA‐treated Vacutainer tubes (BD; Franklin Lakes, New Jersey, USA). Samples were centrifuged at 3000 rpm for 10 min to separate the plasma, which was then aliquoted and stored at −80°C until analysis. To minimize oxidation, carotenoid handling was performed under reduced light, and the samples were processed promptly.

For carotenoid extraction, 200 μL of plasma was mixed with an equal volume of ultrapure water, followed by the addition of 400 μL of ethanol containing an internal standard (Apo‐8′‐carotenal, c = 200 μg/L) for protein precipitation. After vigorous mixing, 800 μL of n‐hexane containing 0.2% butylated hydroxytoluene (BHT) was added. The samples were shaken for 5 min and centrifuged for 2 min at 4°C at 250 × *g*. A total of 400 μL of n‐hexane supernatant was transferred to a 1.5 mL microtube, evaporated to dryness under a stream of argon gas and reconstituted in the mobile phase. The samples were mixed thoroughly before injection into the HPLC system (injection volume: 50 μL).

#### 2.3.2 HPLC Analysis and Carotenoid Quantification

Carotenoid quantification was performed using an Ultimate 3000 HPLC system (Thermo Fisher Scientific, Waltham, MA, USA). The mobile phase consisted of 66% (v/v) acetonitrile, 24% (v/v) methanol, 7% (v/v) tert‐butyl methyl ether, and 3% (v/v) ammonium acetate with L‐ascorbate purchased from Honeywell (Charlotte, North Carolina, USA), Sigma Aldrich (St. Louis, Missouri, USA), and Roth (Karlsruhe, Germany), respectively. The flow rate was set at an isocratic rate of 1.6 mL/min with an approximate runtime of 18 min per sample. A LiChrospher RP‐18 endcapped column (125 × 4 mm, 5 μm particle size; Merck, Darmstadt, Germany) was used as the stationary phase. This column consists of octadecyl‐bonded silica (C18) operated in reversed‐phase mode, which is suitable for separating non‐polar carotenoids using the described mobile phase. Separation of the individual carotenoids, namely, alpha carotene, beta carotene, beta cryptoxanthin, lutein, and lycopene, was achieved based on different retention times of the single compounds. An UV–Vis detector was set at 450 nm for carotenoids and additionally at 472 nm for lycopene. The resulting chromatograms were analyzed using Thermo Scientific Chromeleon Chromatography Data System (Thermo Fisher Scientific, Waltham, MA, USA) software. Carotenoid concentrations were quantified based on calibration curves generated from external standards of individual carotenoids (Carotenature; Münsingen, Switzerland). To ensure analytical accuracy and reproducibility, Standard Reference Material (SRM 968e) from the National Institute of Standards and Technology (NIST) was used.

### 2.4 Cutaneous Carotenoid Measurements

**FIGURE 1:**
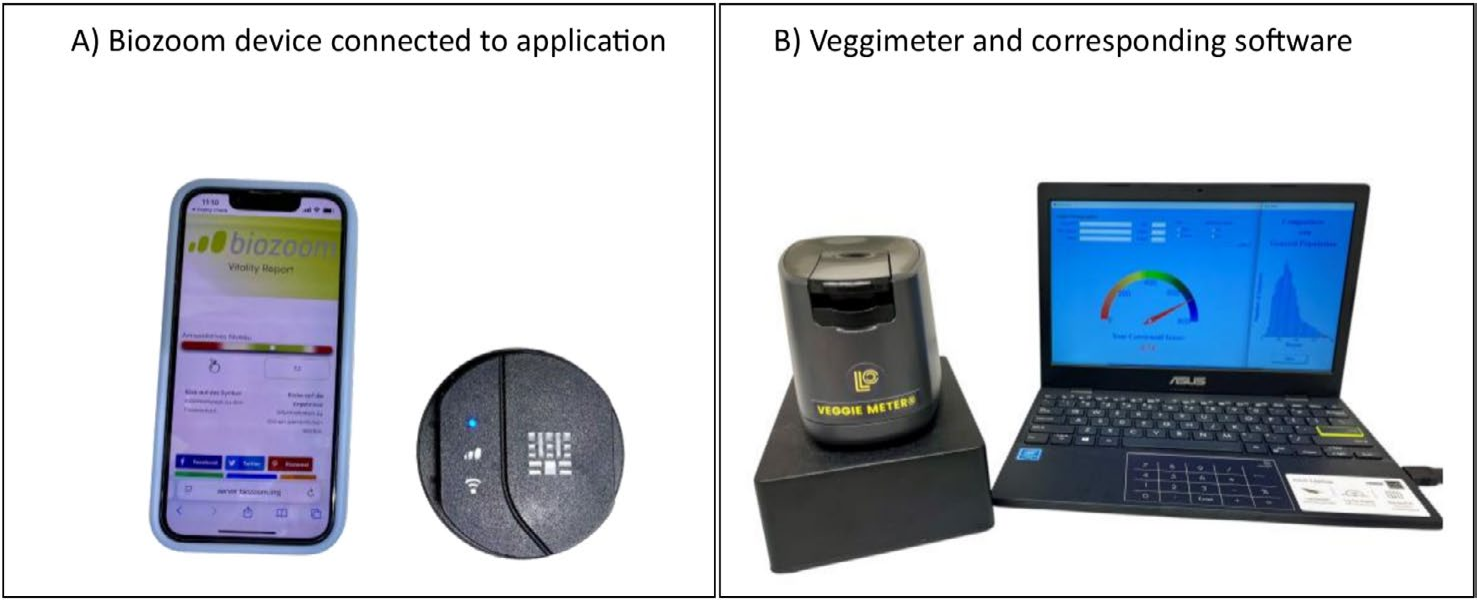
Two spectroscopic devices were used to assess cutaneous carotenoid levels. (A) The Biozoom Scanner, connected to a mobile application, measures carotenoids by having the participant place the palm of the hand along a designated groove on the device. (B) The Veggie Meter, connected to a computer via dedicated software, measures carotenoid levels by inserting a finger into the designated slot. Both devices provide results as Arbitrary Scores, displayed on a continuous scale ranging from low (left) to high (right) carotenoid concentrations. Photographs were taken by the authors.

For determination of cutaneous carotenoid (CC) concentration, two previously validated reflection spectroscopy devices, namely the Veggie Meter (Longevity Link Corporation; Salt Lake City, UT, USA) (Jilcott Pitts et al. 2018) and Biozoom (Biozoom Services; Kassel, Germany) (Matsumoto et al. 2020), were used. Both instruments are based on reflection spectroscopy and measure carotenoids stored in the stratum corneum noninvasively. The devices used are shown in Figure 1.

#### 2.4.1 Veggie Meter

CC levels were assessed via the Veggie Meter. The device was calibrated daily using the provided dark and white reference sticks. For measurement, participants placed their right index finger into the device, and the measurement was initiated. The results were displayed on a connected computer as a score ranging from 0 to 800, with higher values indicating greater carotenoid accumulation in the skin. Additionally, a histogram provided a comparison of individual scores relative to a reference population.

#### 2.4.2 Biozoom

The Biozoom Vitality Check scanner was used as a second method to assess CC levels. The “Vitality Check” application (V0.2.20, Biozoom Services, Kassel, Germany) was installed on a smartphone and connected to the device via Wi‐Fi. No device calibration is required, and after selecting the “nutrition” category, the option “fast measurement” was selected. Participants were instructed to follow on‐screen prompts and place the palm of their hand on the blinking sensor area. Once the sensor was entirely covered, the measurement was initiated. To standardize the procedure, participants were asked to always use the right hand and to clean it prior to the measurement. The measurement takes approximately 10 s, and the results are displayed as values ranging from 0 to 12, with higher values indicating greater carotenoid concentrations in the skin.

### 2.5 Pooled Data Analysis

The primary aim of this study was to examine whether noninvasive cutaneous carotenoid measurements reflect systemic carotenoid status measured by HPLC and whether both are associated with adiposity‐related parameters. Data from the ESAN III study were pooled across three visits (baseline, post‐intervention, and post‐washout), yielding *n* = 151 observations. Although the original study included an intervention phase, treatment effects were not analyzed; instead, pooled data were used to assess overall associations across the population, independent of group allocation or timepoint. While repeated measurements per participant may introduce within‐subject dependencies, correlation analyses were applied to evaluate overall monotonic associations between measurement methods rather than longitudinal changes. This approach is consistent with established validation studies of nutritional biomarkers and spectroscopy‐based cutaneous carotenoid assessment tools and allows to maximize statistical power (Radtke et al. 2020). To further account for repeated measurements, additional analyses were performed using linear mixed‐effect models (LMMs), which is described in detail in Section 2.7 (Statistical Analysis).

### 2.6 Power Analysis

A power analysis was conducted using G*Power software (V3.1, University of Düsseldorf, Germany) (Faul et al. 2009) to determine the sample size required to detect a significant correlation between cutaneous carotenoid levels and body composition parameters. Assuming a medium effect size (*r* = 0.30), an alpha level of 0.05, and a sample size of *n* = 151, a two‐tailed test yielded a critical correlation coefficient of *r* = 0.1598 and a statistical power of 96.54% (1 − β).

### 2.7 Statistical Analysis

Statistical analyses were performed using IBM SPSS Statistics, version 29 (IBM Corporation, Armonk, NY, USA). Continuous variables were assessed for normality using the Shapiro–Wilk test. As several variables were not normally distributed, non‐parametric tests were used for subsequent analyses, and Spearman's rank correlation coefficient was used to evaluate associations between plasma and cutaneous carotenoid concentrations and body composition parameters. This non‐parametric approach does not assume normally distributed data and is robust to outliers. For multiple comparisons, a Bonferroni correction was applied where appropriate.

To account for repeated measurements obtained from the same participants across three study visits, LMMs were applied with participant ID included as a random intercept. Plasma carotenoid concentrations were log‐transformed prior to analysis to improve residual normality and served as the dependent variable. Separate models were fitted for each cutaneous measurement device, with either Biozoom or Veggie Meter values entered as fixed effects to avoid collinearity between devices. An initial unadjusted model was fitted, followed by adjusted models including smoking status (current smoker vs. non‐smoker) and body fat percentage as covariates to assess potential confounding. Regression coefficients from log‐transformed models were interpreted as relative (percentage) changes in plasma carotenoid concentrations. Linear mixed‐effects models were estimated using restricted maximum likelihood (REML). Variance components were used to estimate intraclass correlation coefficients (ICC), representing the proportion of variance attributable to between‐subject differences. Model assumptions, including normality of residuals and homoscedasticity, were assessed using standard diagnostic plots. Statistical significance was defined as *p* < 0.05 unless otherwise specified.

To evaluate whether the strength of the correlations between plasma carotenoids and cutaneous measurements differed between devices, the two dependent correlation coefficients were compared using the Steiger test for overlapping correlations. The test was performed using the online calculator developed by Lee and Preacher (2013) available at http://quantpsy.org.

## 3 Results

### 3.1 Study Population Characteristics

The study population comprised 54 women recruited from Graz and surrounding areas in Austria. The mean age at baseline was 33.1 years. The majority of participants (96.3%) self‐identified as White, and 87.0% reported being non‐smokers. The detailed baseline characteristics are summarized in Table 2. BMI was calculated as weight [kg] divided by height squared [m^2^], yielding a mean BMI of 24.8 kg/m^2^. Among the participants, 43 were classified as normal weight, and 11 as obese.

**TABLE 2:** Characteristics of the study cohort at baseline visit (*n* = 54); the means and interquartile ranges (IQRs) are shown.

Characteristic	Mean (IQR)
Age [years]	33.1 (15)
Height [m]	1.7 (0.1)
Weight [kg]	69.4 (12.9)
Waist circumference [cm]	77.2 (14.0)
BMI [kg/m^2^]	24.8 (4.4)
Total body fat [kg]	24.3 (10.8)
Fat‐free mass [kg]	45.2 (5.6)

	*n* (%)
BMI category	
Normal weight	43 (79.6%)
Obese	11 (20.4%)
Self‐identified ethnicity	
White	52 (96.3%)
Asian	1 (1.9%)
Other	1 (1.9%)
Smoking status	
Current smoker	7 (13.0%)
Not smoking	47 (87.0%)
Intervention group	
Juice	34 (63.0%)
Placebo	20 (37.0%)

### 3.2 Plasma Carotenoid Concentrations

The plasma concentrations of individual carotenoids were quantified via HPLC. Descriptive statistics, including the mean concentrations, standard deviations (SD) and ranges (minimum–maximum), are shown in Table 3. Among the individual carotenoids, beta carotene was the most abundant. The total carotenoid concentration was calculated as the sum of all selected carotenoids, and provitamin A carotenoids (alpha carotene, beta carotene and beta cryptoxanthin) were combined.

**TABLE 3:** Mean concentration and range of individual carotenoids in plasma determined by HPLC and cutaneous carotenoids measured by Biozoom and Veggie Meter. Number of pooled data points, *n* = 151.

Carotenoid	Carotenoid concentration in plasma	
	Mean [μg/L] (SD)	Range, Min–Max [μg/L]
Total carotenoids	1363.58 (535.93)	444.36–3228.74
Provitamin A carotenoids^a^	861.12 (476.16)	136.58–2524.24
Alpha Carotene	173.60 (148.07)	11.20–674.53
Beta Carotene	511.47 (332.41)	37.51–1712.47
Beta Cryptoxanthin	176.05 (123.10)	45.13–1208.86
Lutein	171.60 (80.63)	38.14–445.93
Lycopene	330.86 (133.31)	72.39–819.73

Device	Total cutaneous carotenoids	
	Mean [score] (SD)	Range, Min–Max [score]
Veggie Meter	441 (100)	226–712
Biozoom	5.3 (1.2)	2.8–9.5

^a^ Sum of alpha carotene, beta carotene, and beta cryptoxanthin.

### 3.3 Cutaneous Carotenoid Levels

Cutaneous carotenoid levels were assessed noninvasively via Veggie Meter and Biozoom devices. Veggie Meter yielded scores ranging from 226 to 712, with a mean of 441 ± 100. The Biozoom scores ranged from 2.8 to 9.5, with a mean of 5.3 ± 1.2. The distribution of values was evaluated via the Shapiro–Wilk test. Neither the Veggie Meter (*p* = 0.179) nor the Biozoom (*p* = 0.053) scores significantly deviated from normality, indicating a normal distribution of cutaneous carotenoid levels within the sample.

### 3.4 Associations Between Plasma and Cutaneous Carotenoids

**FIGURE 2:**
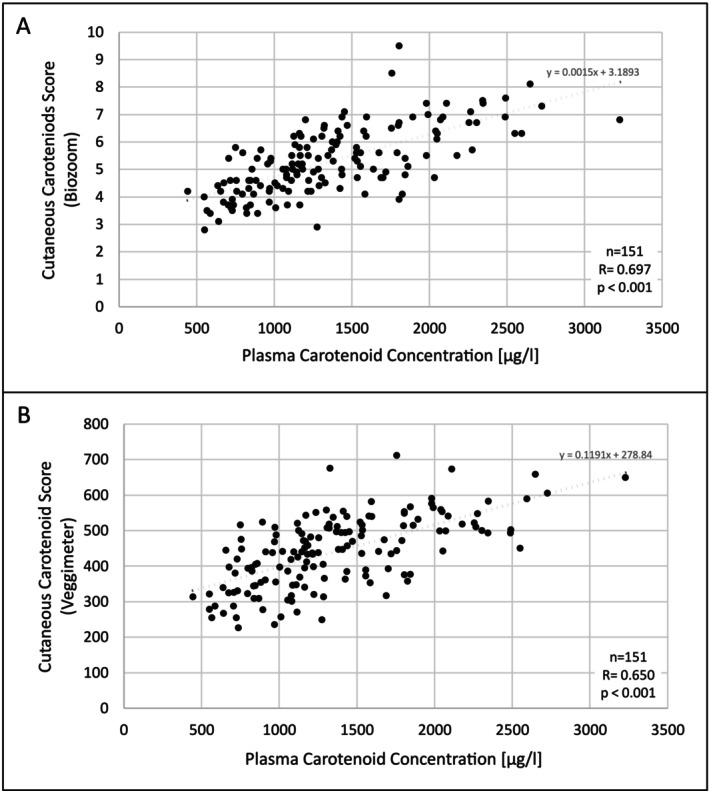
Scatterplots showing the correlation between plasma carotenoid concentration [μg/l] and cutaneous carotenoid scores measured with (A) Biozoom and (B) Veggie Meter.

To assess the relationship between plasma carotenoid concentrations and CC levels, Spearman's rank correlation coefficient was calculated, and scatter plots for Biozoom and Veggie Meter measurements versus total plasma carotenoids were generated (Figure 2). Total plasma carotenoids were significantly correlated (*p* < 0.001) with cutaneous carotenoid levels measured by both devices, with Biozoom showing slightly stronger correlations than Veggie Meter (Biozoom: *r*_s_ (151) = 0.697; Veggie Meter: *r_s_* (151) = 0.650). The correlation between Biozoom and Veggie Meter scores was strong (*r_s_* (151) = 0.725, *p* < 0.001), indicating good agreement of the devices. Comparison of the dependent correlations using the Steiger test indicated a statistically significant difference between the devices (*z* = 0.50, *p* = 0.62).

To account for repeated measurements within participants, the association between cutaneous carotenoid scores and plasma carotenoid concentrations was additionally evaluated using LMMs with participant ID included as a random intercept. The results confirmed the strong positive relationship observed in the correlation analyses. The intraclass correlation coefficient (ICC) was 0.38 for the Biozoom model and 0.29 for the Veggie Meter model, indicating that approximately 29%–38% of the variance in plasma carotenoid concentrations was attributable to between‐subject differences. In the LMMs, cutaneous carotenoid levels measured with Biozoom were significantly positively associated with plasma carotenoid concentrations (*β* = 0.190, *p* < 0.001), corresponding to an approximate 21% increase in plasma carotenoid concentrations per unit increase in Biozoom values. Veggie Meter scores showed a similarly strong positive association (*β* = 0.002, *p* < 0.001), corresponding to an approximate 0.2% increase per unit increase, or approximately 22% per 100‐unit increase in Veggie Meter scores.

Together, these analyses demonstrate that CC measurements obtained with both devices reliably reflect systemic carotenoid status and that this relationship remains robust when accounting for repeated measurements, with additional adjustment for potential confounders presented in Section 3.6.

### 3.5 Associations Between Individual Plasma Carotenoids and Cutaneous Measurements

**FIGURE 3:**
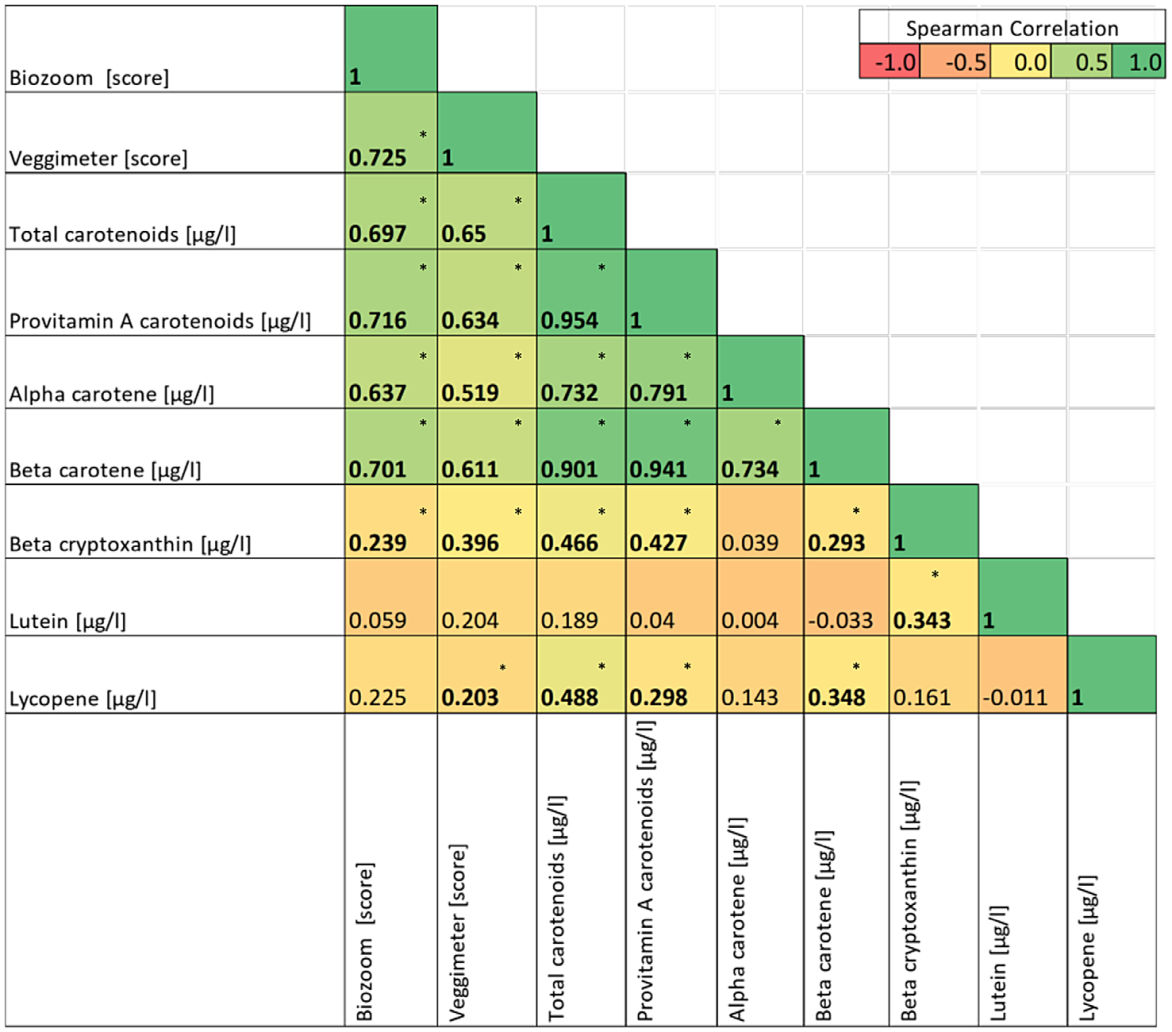
Correlation heatmap of plasma carotenoid (individual and total) levels quantified via high‐performance liquid chromatography (HPLC) and cutaneous carotenoid levels determined via Biozoom and Veggie Meter. Spearman correlation coefficients are presented. The strength and direction of the correlations are indicated by color intensity, as shown at the scale at the top right. Asterisks (*) denote statistically significant correlations after Bonferroni correction for multiple comparisons (adjusted *α* = 0.00139).

Furthermore, Spearman correlations for selected carotenoids were computed between all variable pairs (36 tests). To adjust for multiple comparisons, a Bonferroni correction was applied (adjusted *α* = 0.00139). Correlations with *p*‐values below this threshold were considered statistically significant. The correlation coefficients are summarized in Figure 3. Among carotenoids, beta carotene demonstrated the strongest and most consistent correlations (Biozoom: *r_s_* (151) = 0.709, *p* < 0.001; Veggie Meter: *r_s_* (151) = 0.611, *p* < 0.001). Alpha carotene also correlated moderately to strongly and significantly with both devices (Biozoom: *r_s_* (151) = 0.637; *p* < 0.001; Veggie Meter: *r_s_* (151) = 0.519, *p* < 0.001). In contrast, lycopene was weakly correlated (Biozoom: *r_s_* (151) = 0.225, *p* = 0.005; Veggie Meter: *r_s_* = 0.203, *p* = 0.013), and lutein was weakly correlated (Biozoom: *r_s_* = (151) 0.059, *p* = 0.469; Veggie Meter: *r_s_* (151) = 0.204, *p* = 0.012). The sum of the provitamin A carotenoids (β‐cryptoxanthin, α‐carotene, and β‐carotene) also showed strong positive correlations with the Biozoom (*r_s_* (151) = 0.716, *p* < 0.001) and Veggie Meter (*r_s_* (151) = 0.637, *p* < 0.001) scores. While strong associations were observed for total carotenoids, the strength of the relationship varied substantially across individual carotenoids.

### 3.6 Influence of Body Composition and Smoking on Plasma‐Skin Associations

To evaluate the potential confounding effect of body fat percentage, partial correlation analyses were conducted, controlling for body fat. While the strength of the associations between plasma and cutaneous carotenoid concentrations was slightly attenuated, the correlations remained statistically significant (Veggie Meter partial *r*_*s*_ = 0.574, *p* < 0.001; Biozoom partial *r*_*s*_ = 0.641, *p* < 0.001). These findings indicate that the investigated noninvasive measurements of the CC are robustly associated with systemic carotenoid status, independent of body fat percentage.

To further assess the robustness of the observed associations to potential confounding factors, additional LMMs were performed including smoking status and body fat percentage as covariates. Consistent with the unadjusted models, cutaneous carotenoid levels measured with Biozoom were significantly positively associated with plasma carotenoid concentrations (*β* = 0.183, *p* < 0.001), corresponding to an approximate 21% increase in plasma carotenoid concentrations per unit increase in Biozoom values.

Similarly, Veggie Meter scores remained significantly associated with plasma carotenoid concentrations after adjustment for smoking status and body fat percentage (*β* = 0.002, *p* < 0.001), further confirming the robustness of the observed associations after accounting for potential confounders.

### 3.7 Associations Between Carotenoid Status and Adiposity Measures

**FIGURE 4:**
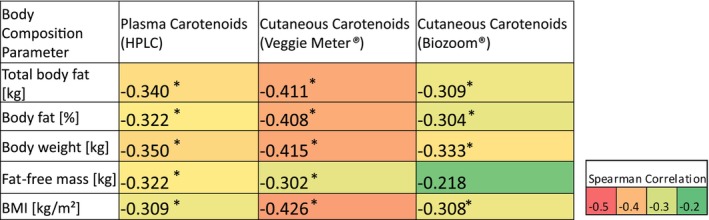
Heatmap of Spearman correlation coefficients of body composition parameters and carotenoid levels in plasma and skin (cutaneous) (*n* = 151). For multiple comparisons, Bonferroni correction was applied, and correlations with *p* < 0.0033 are marked as significant (*).

Both plasma and cutaneous carotenoid levels were inversely associated with body composition parameters related to obesity. Higher total body fat, body fat percentage, body weight, and BMI were significantly correlated with lower carotenoid concentrations. Most correlations had *p*‐values < 0.001 and remained statistically significant after the Bonferroni correction (adjusted *α* = 0.0033). The detailed correlation coefficients are provided in Figure 4. When comparing the two devices, Veggie Meter demonstrated slightly higher negative correlations with adiposity measures than Biozoom, suggesting greater sensitivity to body composition differences.

## 4 Discussion

This study demonstrated strong agreement between plasma carotenoid concentrations quantified by HPLC and cutaneous carotenoid (CC) levels assessed using two reflection spectroscopy devices. Both Veggie Meter and Biozoom showed robust correlations with systemic carotenoid status, particularly for provitamin A carotenoids, which represent major carotenoid species present in human skin (Darvin et al. 2022). Importantly, the association between cutaneous carotenoid measurements and plasma carotenoid concentrations remained significant in LMMs adjusting for smoking status and body fat percentage, indicating that the observed relationships were robust across differences in body composition and lifestyle factors, such as smoking. Together, these findings support the validity of the two investigated noninvasive skin carotenoid measurement devices, supporting their use as practical biomarkers of systemic carotenoid exposure and habitual fruit and vegetable intake in line with previous studies (Darvin et al. 2016; Hwang et al. 2023). From a food and nutrition perspective, these findings are particularly relevant for studies evaluating fruit‐ and vegetable‐rich dietary patterns, functional foods, and carotenoid‐containing products, where objective assessment of dietary exposure is needed.

Comparing HPLC with noninvasive techniques highlights complementary strengths and limitations. HPLC remains the gold standard for carotenoid quantification due to its high analytical specificity and ability to detect individual compounds, including colorless carotenoids such as phytoene and phytofluene, which are not detectable by optical methods but may have biological relevance (Mapelli‐Brahm and Meléndez‐Martínez 2021). However, the invasive nature, cost, and laboratory requirements of HPLC limit its applicability in large‐scale nutritional surveys, community‐based interventions, and studies involving vulnerable populations.

In contrast, reflection spectroscopy–based devices such as the Veggie Meter and Biozoom offer rapid, cost‐effective, and user‐friendly alternatives for estimating carotenoid status. Their portability and ease of use make them well suited for public health and clinical nutrition settings, where they can support monitoring of fruit and vegetable intake and overall dietary quality. Consistent with earlier reports, our findings confirm that CC measurements reflect longer‐term carotenoid exposure and habitual plant food consumption (Rush et al. 2020).

Importantly, CC levels appear to integrate dietary exposure over longer time frames than plasma carotenoids, which respond more rapidly to short‐term dietary changes. Meinke et al. (2010) demonstrated that plasma carotenoid concentrations change more quickly than skin levels following dietary interventions (Meinke et al. 2010), a finding further supported by Radtke et al. (2020), who reported delayed increases in CC relative to plasma carotenoids (Radtke et al. 2020). Thus, CC measurements may be particularly valuable as indicators of habitual fruit and vegetable intake, whereas plasma carotenoids may be preferable for assessing acute dietary responses or bioavailability.

While strong associations were observed for total carotenoids, the strength of the relationship between plasma and cutaneous measurements differed across individual carotenoids. Beta carotene showed the strongest correlations, whereas lycopene and lutein exhibited weaker relationships. Lycopene's high lipophilicity promotes sequestration in adipose tissue, which serves as an important storage site for carotenoids and may reduce circulating plasma concentrations (Landrier et al. 2012; Matsumoto et al. 2020). Lutein, in contrast, belongs to the more polar xanthophyll class, resulting in differences in lipoprotein transport and tissue distribution compared with carotenes. Moreover, lutein preferentially accumulates in ocular tissues, particularly the macula of the retina, rather than in the skin, which may weaken the relationship between plasma concentrations and cutaneous measurements (Flieger et al. 2024; Krinsky and Johnson 2005). Beyond these biological factors, methodological characteristics of optical measurement techniques may also contribute to the observed differences in correlation strength among individual carotenoids. Reflection spectroscopy detects carotenoids within a specific optical window in the visible spectrum (approximately 460–520 nm), where carotenoid chromophores exhibit strong absorption and interference from other skin chromophores is relatively limited (Ermakov et al. 2018). Because individual carotenoids possess distinct absorption spectra determined by their conjugated double‐bound structures, their contribution to the reflected optical signal varies, with beta carotene typically representing the dominant contributor to skin carotenoid signal detected by spectroscopy‐based methods (Darvin et al. 2016).

Higher BMI and total body fat were consistently associated with lower plasma and cutaneous carotenoid levels, supporting extensive evidence that individuals with obesity exhibit reduced carotenoid status—even when dietary intake is similar to that of normal‐weight individuals (Calder et al. 2011; Hamulka et al. 2023). In line with these observations, our mixed‐effects models also indicated that higher body fat percentage was negatively associated with plasma carotenoid concentrations. Several mechanisms likely contribute to this observation, including increased oxidative stress and chronic low‐grade inflammation, which increase utilization of antioxidants, as well as sequestration of carotenoids in adipose tissue, reducing their circulating concentrations.

In addition, differences in carotenoid bioavailability may further contribute to this “adiposity gap,” as carotenoid absorption depends on lipid digestion, micelle formation, and intestinal transport processes that can vary between individuals (Bohn et al. 2021; Coronel et al. 2019). Moreover, carotenoids may also influence adipose tissue biology through the modulation of PPAR‐γ signaling and lipid oxidation pathways (Bohn et al. 2021). Evidence from weight loss interventions further supports this relationship, demonstrating that reductions in fat mass correspond to increases in plasma carotenoid concentrations, suggesting dynamic interactions between systemic availability and tissue storage (Hamulka et al. 2023; Lackner et al. 2021). Collectively, these findings underscore the importance of considering body composition when interpreting both plasma and cutaneous carotenoid biomarkers.

Several limitations should be acknowledged. The study population was relatively homogeneous, consisting exclusively of women with limited ethnic diversity, which may restrict the generalizability of findings to more diverse populations. Because optical methods rely on the interaction of light with skin chromophores, variations in skin pigmentation may theoretically influence measurement accuracy. However, the potential impact of melanin on the present measurements is likely limited for several reasons. First, measurements were performed at anatomical sites with naturally low pigmentation, the palm of the hand for the Biozoom device and the fingertip for the Veggie Meter. Palmar and fingertip skin contain substantially lower melanin levels than most other body areas and show relatively small variation in pigmentation across different skin types, which is why these regions are commonly recommended sites for reflection‐spectroscopy–based carotenoid measurements (Darvin et al. 2012; Ermakov et al. 2018).

Second, the optical principles of both devices are specifically designed to detect carotenoids based on their characteristic absorption and scattering properties associated with their long conjugated double‐bond structures, which produce distinct optical signals in the visible wavelength range (Darvin et al. 2022). Carotenoids exhibit strong absorption in the 460–520 nm spectral range, which represents an optical window with relatively limited overlap with other skin chromophores such as hemoglobin and melanin (Ermakov et al. 2018). Furthermore, modern reflection spectroscopy approaches incorporate spectral deconvolution algorithms that quantify and account for contributions from other chromophores, including melanin and hemoglobin, as well as tissue scattering when calculating carotenoid scores. Empirical studies evaluating these algorithms in populations with a wide range of skin pigmentation have shown no significant association between measured skin carotenoid scores and independently measured melanin indices, indicating that higher melanin levels do not systematically bias carotenoid measurements (Ermakov et al. 2018). Similarly, validation studies across racially and ethnically diverse populations have demonstrated robust associations between skin carotenoid measurements and dietary intake or plasma carotenoid concentrations, supporting the overall reliability of these optical methods (Jilcott Pitts et al. 2018; Obana et al. 2023).

Nevertheless, the limited ethnic diversity of the present cohort should still be acknowledged when considering the broader applicability of the findings. Future studies including participants representing a wider range of Fitzpatrick skin types will be important to further confirm the generalizability of noninvasive carotenoid assessment across diverse populations.

Another methodological consideration relates to the anatomical sites used for carotenoid measurements. The Veggie Meter assesses carotenoid levels at the fingertip, whereas the Biozoom device measures the palm of the hand. Structural and biochemical characteristics of the skin vary across anatomical regions, including differences in stratum corneum thickness, lipid composition, and barrier functions (Mohammed et al. 2012), which could potentially influence optical carotenoid measurements. However, our data suggest that the different anatomical measurement sites likely do not substantially influence the observed relationships in plasma and cutaneous carotenoid levels.

Additionally, while pooling repeated measurements increased statistical power, repeated observations may introduce within‐subject dependencies. To address this, we applied LMMs including participant ID as a random effect, which confirmed the robustness of the associations between plasma and cutaneous carotenoid levels. Future studies incorporating longitudinal designs and broader demographic representation would further strengthen the validation of these noninvasive carotenoid assessment methods.

Overall, this study supports the practical utility of noninvasive CC assessment as a scalable nutritional biomarker. Reflection spectroscopy–based devices enable rapid, objective monitoring of fruit and vegetable intake and may enhance dietary assessment in nutrition research, clinical practice, and public health interventions aimed at preventing non‐communicable diseases (Boeing et al. 2012). While both devices provided valid estimates of systemic carotenoid status, the Veggie Meter showed slightly stronger associations with adiposity‐related measures, whereas the Biozoom offers advantages in portability and user‐friendly operation.

In conclusion, this study demonstrates that cutaneous carotenoid measurements obtained using reflection spectroscopy provide a robust and noninvasive biomarker of systemic carotenoid status. Strong associations between cutaneous carotenoid scores and total plasma carotenoid concentrations were observed for both devices, supporting the validity of these methods for assessing carotenoid exposure. Importantly, these relationships remained significant after accounting for repeated measurements, smoking status, and differences in body composition, indicating that cutaneous carotenoid measurements can reliably reflect systemic carotenoid levels across individuals with varying adiposity and lifestyle factors. However, the strength of the associations differed substantially among individual carotenoids, with provitamin A carotenoids showing stronger correlations than lutein and lycopene. These findings highlight both the utility and the limitations of optical carotenoid measurements and should be considered when interpreting cutaneous carotenoid scores in nutrition research and dietary assessment.

## Author Contributions

**Franziska Kiem:** investigation, visualization, writing – original draft, formal analysis. **Sabrina Mörkl:** writing – review and editing. **Nathalie Meier‐Allard:** methodology, investigation, formal analysis. **Christa Schimpel:** writing – review and editing. **Tina Unterweger:** investigation. **Jan Gruber:** investigation. **Sandra Holasek:** conceptualization, supervision, writing – review and editing. **Herbert Strobl:** resources. **Sonja Lackner:** conceptualization, writing – review and editing, supervision.

## Funding

The authors have nothing to report.

## Ethics Statement

The study was approved by the Ethics Committee of the Medical University of Graz (No. 36‐273 ex 23/24) and conducted in accordance with the Declaration of Helsinki.

## Consent

All participants provided written informed consent.

## Conflicts of Interest

The authors declare no conflicts of interest.

## Data Availability

The datasets generated and/or analyzed during the current study are available from the corresponding author upon reasonable request.
